# Mosè Barabba: A case study between forensic and cultural psychiatry

**DOI:** 10.1192/j.eurpsy.2025.230

**Published:** 2025-08-26

**Authors:** J. Santambrogio, Antonino Giancontieri, Elisabetta Leon, Elena Miragliotta, Enrico Capuzzi, Fabrizia Colmegna, Massimo Clerici

**Affiliations:** Disability Unit, ASST Brianza, Limbiate, Italy

## Abstract

**Abstract:**

The presentation gives a narrative account of a case of ethnopsychiatry and criminology from the standpoint of the care network, with personal details supplied by the patient himself. The person involved, Mosè Barabba, was born in Sierra Leone where he was kidnapped and forced to become a child soldier at a very early age. He was brainwashed, brutalized and taught to admire and practice violence. He arrived in Italy at 16 years of age after a traumatic journey through North Africa, homeless and alone. At 19 years old, he was hospitalized for drug abuse, and then cared for by various associations (NAGA, CPS, SPDC). The presentation outlines his descent into substance abuse-induced violence and the first encounter with the law when he was twenty-five years old, followed by a return to a criminal environment after a first experience in a community. He was diagnosed with PSTD, NOS psychosis, behavioral issues related to substance abuse and chronic multifactorial hepatopathy. By the time he was 28 his violence had spiraled out of control and he was hospitalized by TSO, where the violence continued. He was committed to a two-year period in a REMS 1 , for psychopharmacological therapy with mood stabilizers. On his release from the REMS, he was placed in a rehabilitation program in AS.FRA 2 where positive results were obtained: his pathology was stabilized, with support he was able to abstain from substances and alcohol and he was receptive to therapy and rehabilitation. In 2021, then 31 years old, Mosé was considered to be well integrated into the community and was transferred to a CPA (a high protection residence for patients with psychiatric diagnosis).

**Disclosure of Interest:**

None Declared

